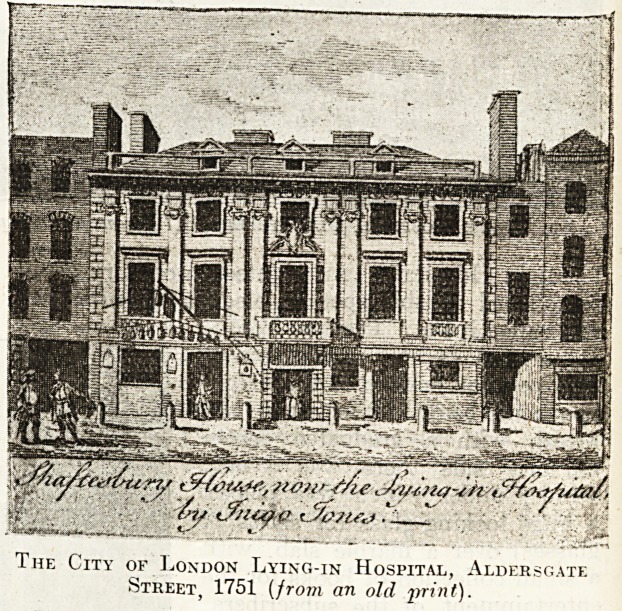# The City Road

**Published:** 1921-04-16

**Authors:** 


### HOSPITAL NEIGHBOURHOODS.

### II. The City Road.

Up and down the City Road,
In and out the Eagle,
That's the way the money goes,
Pop goes the weasel.

It is not ours to dwell upon the incident of the weasel. We have heard of rabbits popping into burrows, and we might consider the possibility of weasels doing something of the kind. It is to be feared, however, that the operation mentioned in the mid-Victorian song has a more sophisticated meaning.

The City Road was in the eighteenth century called the New Road, and then, as now, the first of that continuous string of important thoroughfares carrying the traveller as far as Paddington. North of its junction with Old Street there was formerly a dangerous pond called the "Perilous Pool." Its location was near the back of the site where now stands the City of London Maternity Hospital (formerly the City of London Lying-in Hospital). An eighteenth-century genius established on this spot an elegant pleasure bath, and called it the "Peerless Pool." It must have been an important and popular feature of the neighbourhood, being 170 feet long, 100 broad, with a graduated depth. On one side there was a neat arcade, under which was placed a huge looking-glass (or series of glasses) over a marble slab, with a small collection of books for the entertainment of the subscribers. There were other baths, pleasure grounds, and a fishing pool.

St. Luke's Hospital, Old Street, 1817 (*from an old print*).

The Peerless Pool must have survived until early in the nineteenth century, when we find a contemporary writer lamenting the approaching disappearance of "this useful appendage to the Metropolis," and the fact that it "will shortly give way to the rage of improvement which has opened itself to this neighbourhood and be superseded by low, mean rows of houses."

St. Luke's Hospital, Upper Moorefields, 1768 (*from an old print*).

Thus may have commenced the departure of gaiety from the City Road district, and with the passing of the Eagle Music-hall, mentioned in the prefacing verse, complete solemnity may be said to have come to stay.

As St. Luke's Hospital was opened at the City Road end of Old Street in 1787, and the Lying-in Hospital appeared round the corner in 1772, the Peerless Pool must have been not far from the grounds of both these institutions. St. Luke's (like Bethlem and the Royal London Ophthalmic) came from Moorfields, which may be described as an extinct hospital district. It was opened at the top of Windmill Hill in 1751, and after thirty-eight years was found to be inadequate for its requirements. We are able to reproduce views of the original hospital, and the rugged and rather gloomy-looking building erected in Old Street by Mr. George Dance and opened in the year 1787.

The French Hospital, near Old Street, about 1800 (*from an old print*).

The previous article appeared on April 2.

**Hospital Neighbourhoods**—(*continued*)

During recent excavations in Old Street the foundation-stone of 1787 was discovered. The inscription is in the form of an engraved copper plate, encased in a leaden sealed envelope, fitted in between two massive blocks of stone.

The St. Luke's building still stands where it did, but the work is in abeyance. The lease was sold to the Bank of England at the end of 1916, and

The City of London Lying-in Hospital, City Road, 1772 (*from an old print*).

the famous mental institution now seeks a home in a more rural district.

In Pesthouse Row, near Old Street Square, was erected in 1717 the French Hospital, now situated in Hackney. Letters patent were granted by George I., and the governors were constituted a body politic and corporate by the name of "The Governors and Wardens of the Hospital for Poor French Protestants and their Descendants residing in Great Britain." Of the distinctly Huguenot institutions in England few have had a more interesting history or a more useful existence than this. It is, of course, more an almshouse than a hospital, and is not to be confused with the other excellent institution of the same name in Shaftesbury Avenue, which is, of course, a hospital in the modern sense.

We next come to the City of London Maternity Hospital, instituted in 1750 in Aldersgate Street for "married women and sick and lame out-patients." Shortly afterwards Inigo Jones's building, called Thanet or Shaftesbury House, was taken and fitted up for the purposes of the charity, and the work was restricted to married women only, and the name changed to the "City of London Lying-in Hospital for Married Women." Shaftesbury House, of which there are a number of engraved views, must have been a remarkable building, worthy of the illustrious designer of the Banqueting Hall at Whitehall.

In 1772 the hospital was transferred to the present site, and in 1904, in consequence of the damage to the foundations and structure caused by the construction of the tube railway near by, the building was condemned by the London County Council as unsafe. The cupola, supported by four columns, shown in the engraving of 1772, disappeared many years ago. The latest building was opened in 1907, and its chapel in 1910.

The next comer to this hospital neighbourhood was St. Mark's Hospital, which, to meet the growing demands of poor persons suffering from fistula, etc., was removed here from Charterhouse Square, and occupied the adapted buildings of Dyer's Almshouses towards the Islington end of City Road. The permanent building was erected in 1894-95, where the merciful work of this splendid charity is vigorously carried on.

The last arrival was the Royal London Ophthalmic Hospital, otherwise known as Moorfields Eye Hospital. This institution was founded in 1804, and was first to be found in Charterhouse Square. Some of the first patients were soldiers and sailors invalided home from Sir Ralph Abercrombie's Egyptian expedition.

Expansion becoming necessary, the hospital removed about 1821 to Lower Moorfields, when the building next door to the Roman Catholic church and adjoining Broad Street and Liverpool Street Stations, still so well remembered by a number of us, was built. There are several engravings of the hospital during this part of its history. The present building is in what was known as "Finsbury Fields North," where were at one time great archery grounds, mentioned in a work on archery by W. Hole (about 1600), now in the Bodleian, and containing a map indicating the position of the Perilous (or, later, Peerless) Pool.

The magnificent public service rendered by the Royal London Ophthalmic Hospital in its long history is too well known for extended mention here.

The City of London Lying-in Hospital, Aldersgate Street, 1751 (*from an old print*).

There is yet one more City Road hospital to mention—the Royal Hospital for Diseases of the Chest, established in 1814 and rebuilt in 1863. This is claimed to be the first hospital established in Europe for the study and treatment of consumption and is to be amalgamated with the Great Northern Central Hospital.

## Figures and Tables

**Figure f1:**
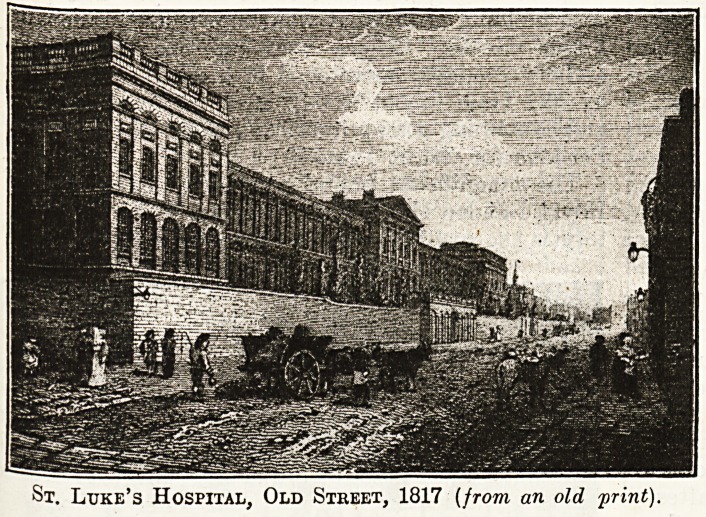


**Figure f2:**
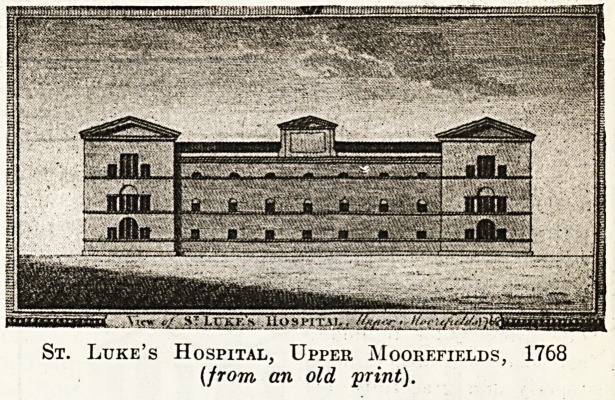


**Figure f3:**
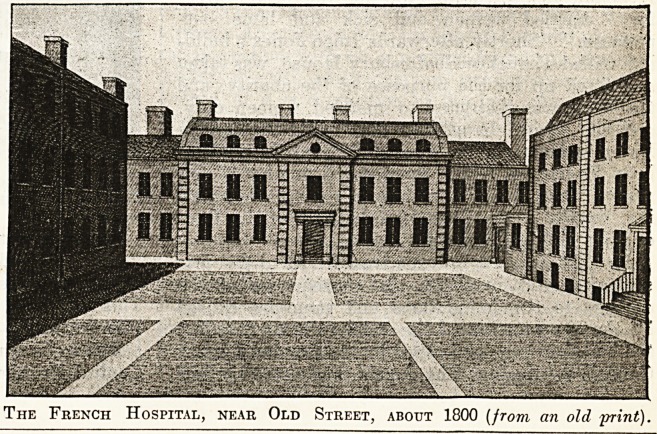


**Figure f4:**
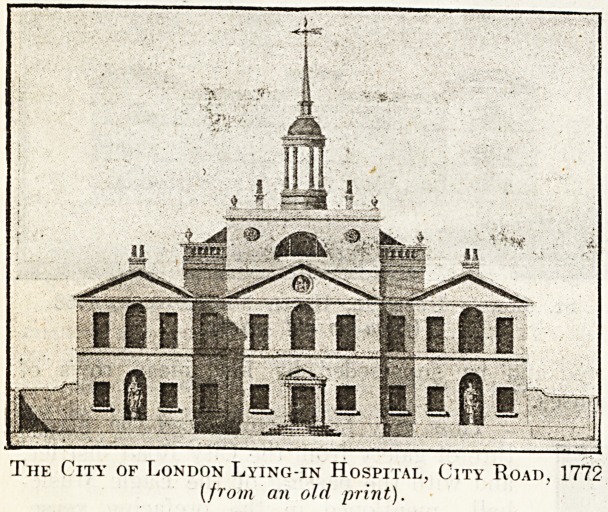


**Figure f5:**